# Saudi Patient-Derived Brain and Intracranial Explanted Tumor Cells: Isolation, Growth, and Anticancer Drug Screening

**DOI:** 10.3390/ijms27167139

**Published:** 2026-08-09

**Authors:** Saba M. Alsubaie, Rafa Almeer, Ali H. Alassiri, Ahmed Alkhani, Fahd AlSufiani, Imadul Islam, Mohamed Boudjelal, Rizwan Ali

**Affiliations:** 1 King Abdullah International Medical Research Center (KAIMRC), King Saud Bin Abdulaziz University for Health Sciences, Ministry of National Guard Health Affairs, Riyadh 14811, Saudi Arabia; ranyasms@windowslive.com (S.M.A.);; 2 Zoology Department, King Saud University, Riyadh 11451, Saudi Arabia; 3 Department of Pathology and Laboratory Medicine, King Abdulaziz Medical City, Ministry of National Guard—Health Affairs, Riyadh 11426, Saudi Arabia; 4 Division of Neurosurgery, Department of Surgery, King Abdulaziz Medical City, Ministry of National Guard—Health Affairs, Riyadh 11426, Saudi Arabia

**Keywords:** brain tumor, drug treatment, microscopy, quinoline-based compounds

## Abstract

Brain cancer is a highly aggressive disease with limited treatment options, highlighting the need for reliable preclinical models for drug discovery. This study aimed to isolate and characterize Saudi patient-derived primary brain cancer cells and assess the anticancer activity of novel compounds developed in-house. Sixteen tumor samples from Saudi patients were processed to establish primary brain cancer cultures and One Normal Tissue (Control). The cells were successfully isolated and maintained under optimized conditions, with their morphology and growth characteristics monitored. Molecular analysis confirmed the expression of key tumor and neural markers. The anticancer activity of selected compounds, KCO69, KCO70, and KCO129, was tested at various concentrations using the MTT and CellTiter-Glo Luminescent Cell Viability Assay. All compounds caused a concentration-dependent reduction in cell viability, with the strongest effects seen at 25 µM. Among them, compound 70 showed the most significant antiproliferative activity, while compounds KCO69 and KCO129 exhibited moderate effects. Variability in treatment response among cultures reflected the inherent heterogeneity of patient-derived tumors. Overall, establishing primary brain cancer cell models from Saudi patients offers a valuable platform for preclinical drug screening and supports further research on these compounds as potential therapies for brain cancer.

## 1. Introduction

Cancer has become one of the most significant global health challenges, with its incidence increasing from the 20th to the 21st century. Current data show that about one in four people is at risk of developing cancer during their lifetime, highlighting the urgent need for ongoing research and effective prevention strategies, especially scientific research based on primary human cancer cells [1].

Brain cancer encompasses malignant conditions affecting the brain, cranial nerves, spinal nerves, spinal cord, and meninges. These cancers are inherently primitive, invasive, and aggressive, often resulting in low survival rates following diagnosis. Brain tumors are categorized by their nature, origin, growth velocity, and stage of progression, and can be classified as benign or malignant [2]. The World Health Organization (WHO) assigns these tumors into four grades (I–IV) based on their aggressiveness. Common examples include metastatic brain tumors, meningiomas, astrocytoma, and glioblastomas. Notably, glioblastoma is among the most aggressive primary brain tumors, characterized by its infiltration into surrounding brain tissue and its resistance to treatment. In Saudi Arabia, brain cancer represented 2.8% of all cancer cases in 2014 and 2016, highlighting its significance and the urgent need for effective interventions [3].

The incidence of brain tumors is increasing worldwide, with variations across countries and regions. The highest rates are observed in developed Western nations, while Africa has the lowest rates. The mortality rate for nervous system cancers is estimated at 3.4 per 100,000 globally [4]. Treating brain tumors often involves Temozolomide (TMZ) [5], which is the standard chemotherapy drug. Other common drugs include Lomustine (CCNU) [6], Carmustine (BCNU) [7], and the PCV regimen (Procarbazine, Lomustine, Vincristine) [8]. Targeted therapies such as Bevacizumab (Avastin) [9] and newer IDH inhibitors, like Vorasidenib, are also used [10]. However, new medicines are urgently needed for brain tumors because current standard treatments—primarily surgery, radiation, and chemotherapy (like temozolomide)—often have limited success, especially for aggressive, recurrent, or resistant cancers. Although progress has been slow, recent years have seen the approval of new targeted therapies, with many more in clinical trials, aimed at overcoming challenges such as the blood–brain barrier and tumor heterogeneity.

The focus is now shifting toward personalized medicine, where treatments are designed based on a patient’s tumor’s unique genetic mutations. Clinical trials are actively testing new compounds, such as MT-125, for glioblastoma [11], to replace or complement current, less effective therapies.

Cell lines are valuable research tools for several reasons, offering distinct advantages in various scientific applications. They are cost-effective, easy to use, and can provide an unlimited supply of materials, thereby bypassing ethical concerns about the use of animal and human tissues. While cell lines offer numerous advantages, their limitations and proper usage considerations are critical to ensuring the validity of experimental findings. One major concern is that cell lines do not always accurately mimic primary cells due to genetic modifications and prolonged culture, which can significantly alter their physiological relevance [12]. Phenotypic and genotypic variations arising from serial passage and genetic drift can further impact cell behavior, reducing the reproducibility and reliability of data.

Primary cells, derived directly from animal or human tissues (explants), are used in drug discovery to provide more physiologically relevant data than immortalized cell lines, as they more closely resemble in vivo conditions. However, their lifespan is limited by replicative senescence—an aging process linked to a predetermined number of cell divisions [13].

Cancer drug development is an expensive and inefficient process with high failure rates, mainly due to insufficient efficacy and safety issues identified during clinical trials. A key reason for this attrition is the limited predictive power of traditional preclinical models, which often fail to capture tumor heterogeneity and the influence of the tumor microenvironment. Although newer models such as organoids and xenografts have improved biological relevance, they remain inadequate, particularly for evaluating immunotherapies. Patient-derived explants therefore represent a promising preclinical approach, as they better preserve human tumor complexity and may improve the prediction of clinical drug responses [14]. In this study we use Temozolomide as reference drug. It is an alkylating agent derived from dacarbazine, which is a foundational anticancer therapy. Its effectiveness is due to its favorable pharmacokinetics: it exhibits excellent oral absorption and can cross the blood–brain barrier (BBB). This property is especially important for treating central nervous system malignancies. TMZ was officially approved in 2005 for the treatment of Glioblastoma Multiforme (GBM), and its clinical use has since expanded to include other solid tumors such as advanced neuroendocrine neoplasms [15].

Quinoline was initially isolated as an impure compound from coal tar by Runge in 1834. Subsequently, in 1842, Gerhardt obtained quinoline as a degradation product of quinine and cinchonine, later it was called quinoline [16].

Quinoline is recognized as one of the most important nitrogen-containing heterocyclic scaffolds in medicinal chemistry. The presence of a nitrogen atom within the quinoline ring system contributes significantly to the basicity of quinoline-based compounds and facilitates potential hydrogen-bonding interactions with biological targets, particularly enzymes. In addition, the polarity introduced by the heteroatom can be strategically utilized in drug design to reduce lipophilicity and enhance aqueous solubility, thereby improving oral bioavailability and absorption [17].

**Figure 1:**
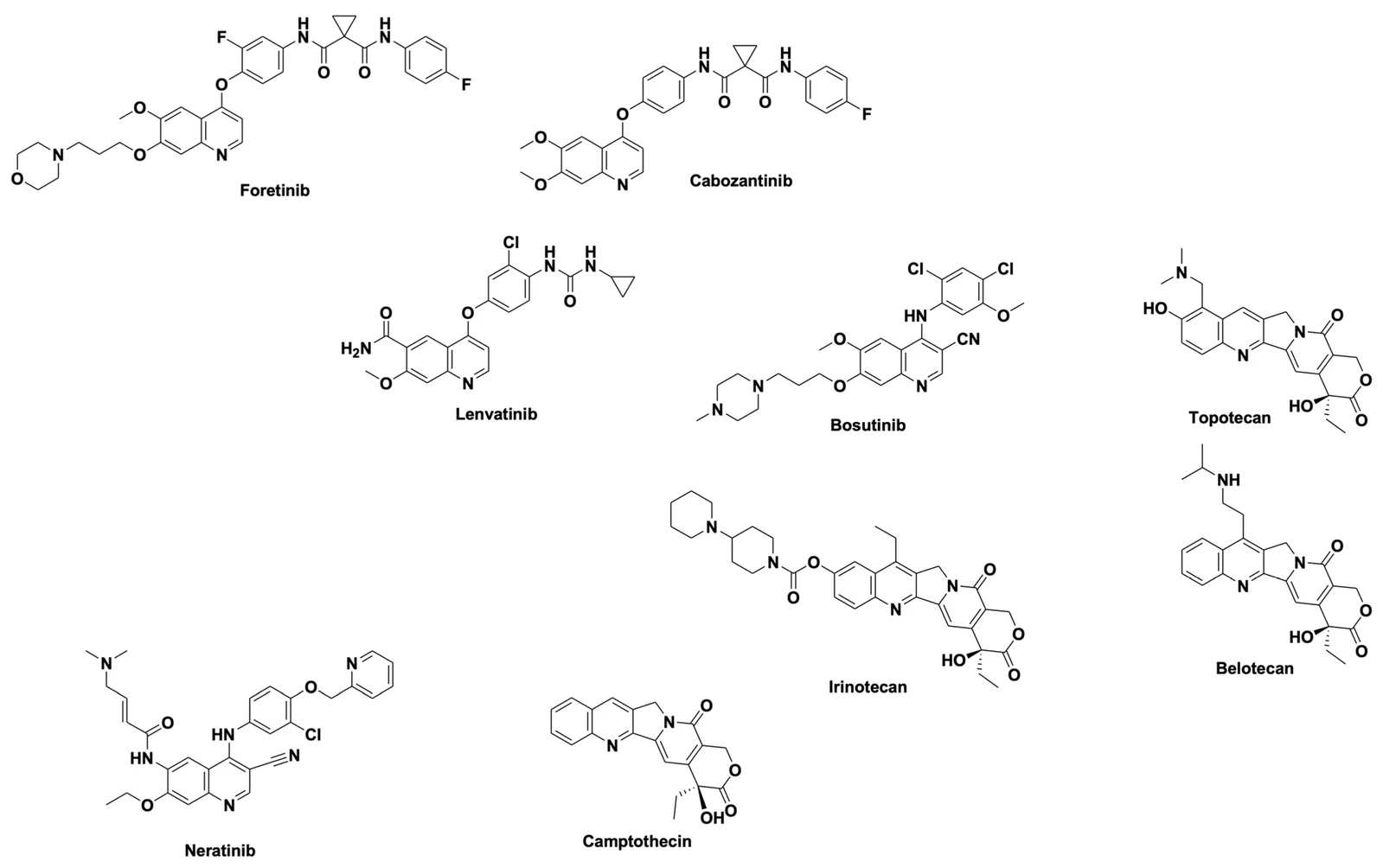
The structures of some approved quinoline-containing anticancer drugs.

The quinoline scaffold exhibits a broad spectrum of biological activities, including anti-HIV, antipsychotic, antibacterial, anti-inflammatory, Phosphodiesterase 4B inhibitory, and antihypertensive effects. Several clinically approved drugs incorporate the quinoline ring system, including Mefloquine, Chloroquine, Quinine, and Amodiaquine, which have been widely used to treat malaria (Figure 1).

Beyond antimalarial therapy, the quinoline nucleus also represents a key structural framework in several marketed anticancer agents. Quinoline derivatives have demonstrated promising anticancer activity through multiple mechanisms of action, including inhibition of tyrosine kinases, alkylation of cellular targets, induction of cell-cycle arrest, suppression of angiogenesis, activation of apoptotic pathways, disruption of cell migration, targeting of the anti-apoptotic protein Bcl-2, and inhibition of antimitotic tubulin polymerization [17].

**Figure 2:**
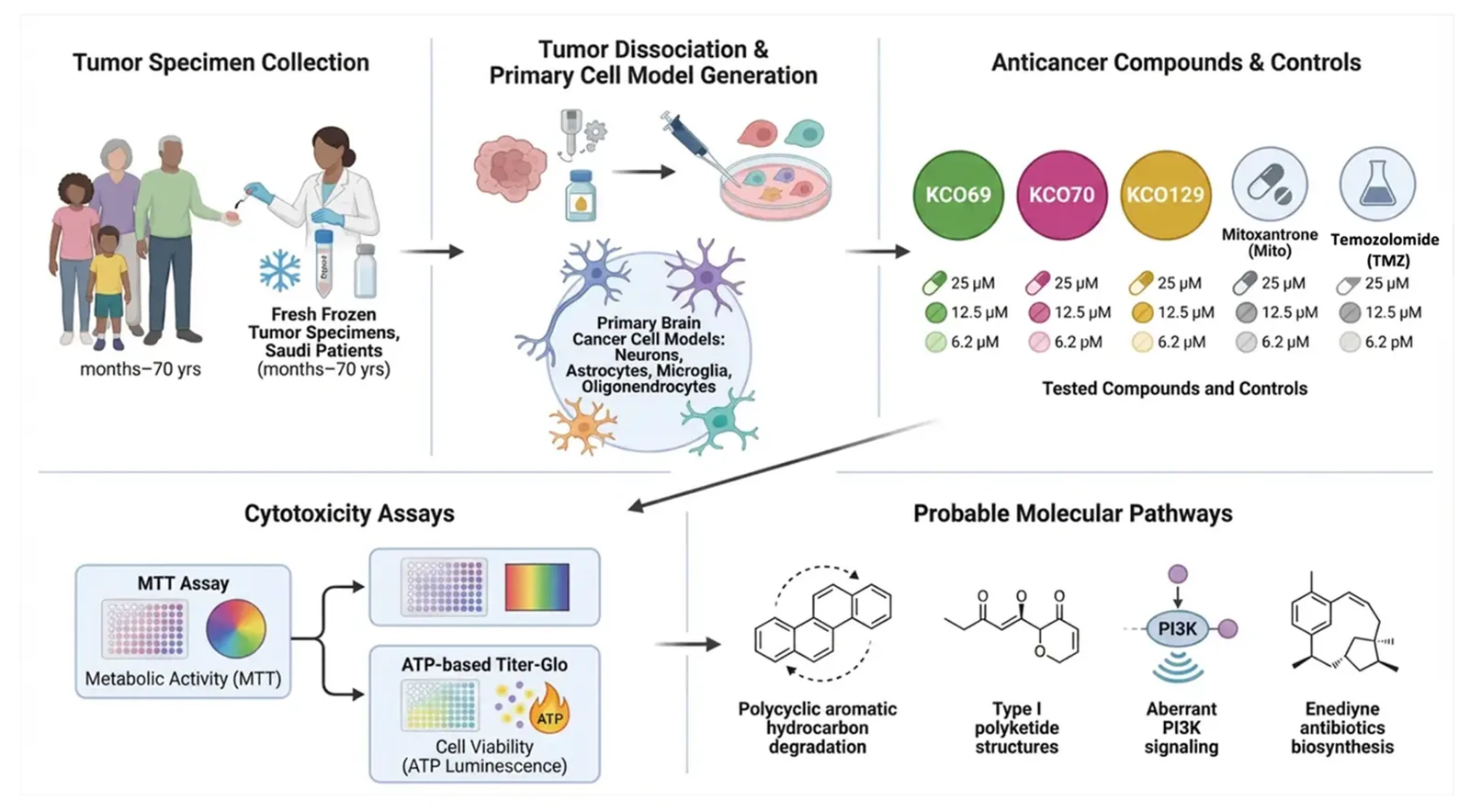
Flow chart of the current study.

These diverse biological properties highlight the significant therapeutic potential of quinoline-based compounds as versatile scaffolds in modern drug discovery and development. We have synthesized a few quinoline-based compounds and tested the promising compound against brain tumor cell lines and patient-derived brain tumor samples, because there is extensive evidence in the literature that gliomas and glioblastomas are morphologically and functionally very heterogeneous tumors, often displaying a complex network of intercellular cross-talks, very difficult to tackle therapeutically due to the insurgence of several parallel mechanisms of resistance. We have identified three compounds that have the potential to significantly affect the growth of brain tumor cells in vitro (Figure 2).

## 2. Results and Discussion

### 2.1. Histology and Pathology

Hematoxylin and Eosin (H&E)-based frozen section (FS) pathology is presently the global standard for intraoperative tumor assessment (ITA). Two cases (P6 as Normal Tissue and P2 as Cancer Tissue) were identified, and their paraffin-embedded blocks were used to generate the hematoxylin–eosin slides.

**Figure 3:**
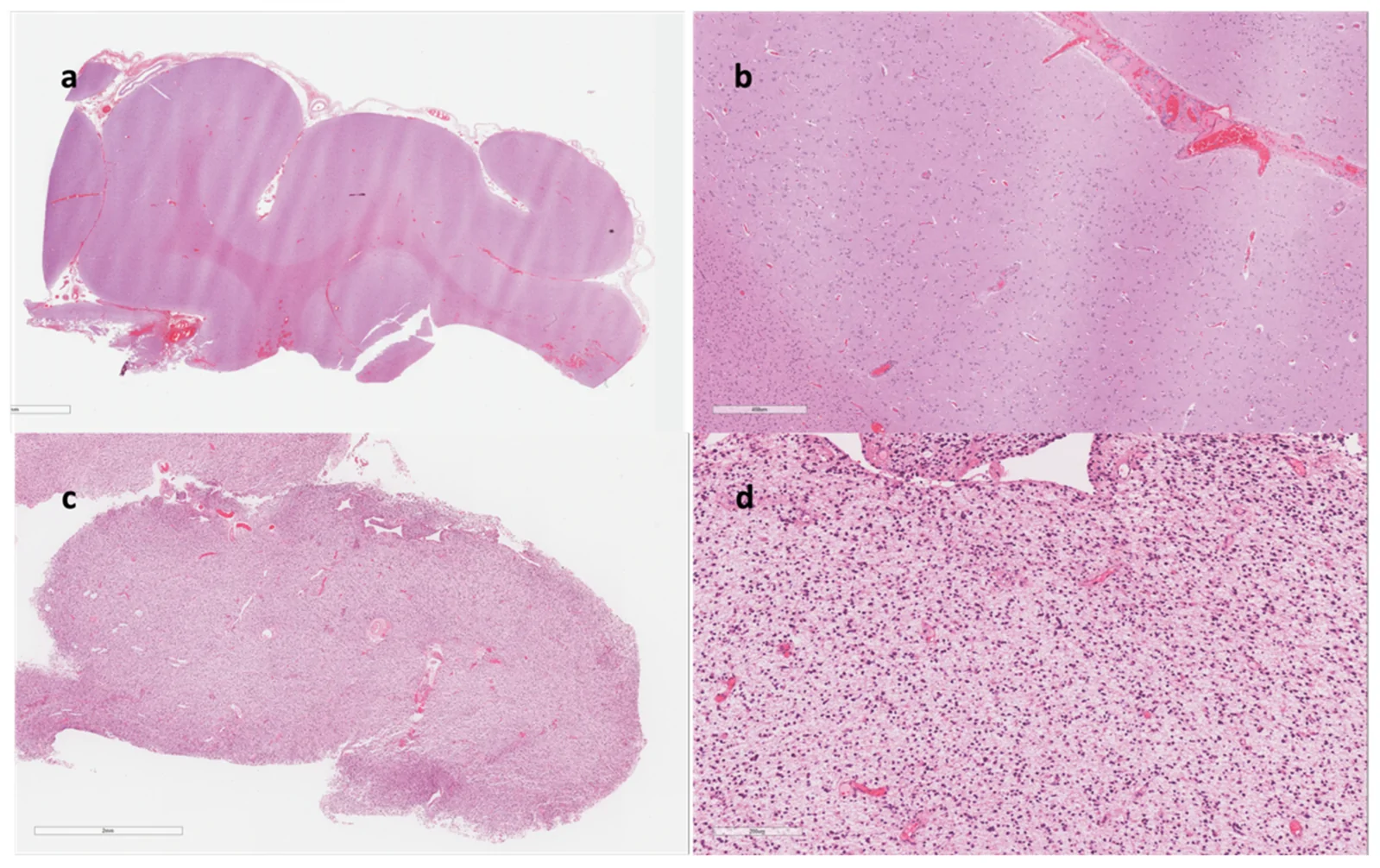
(**a**) Hematoxylin and eosin-stained section from (Normal Tissue-P6), scale bars = 4 mm. (**b**) Hematoxylin and eosin-stained section from (Normal Tissue-P6), scale bars = 40 μm. (**c**) Hematoxylin and eosin-stained section from (Cancer Tissue-P2) High-Grade Astrocytoma, WHO grade 4, scale bars = 2 mm. (**d**) Hematoxylin and eosin-stained section from (P2), scale bars = 200 μm.

Microscopic examination of the H&E-stained sections revealed distinct morphological differences between normal and malignant cerebral tissues. The histological layers of the healthy cerebral cortex in (Normal Tissue-P6) were clearly preserved, as demonstrated in Figure 3a,b. The cortical laminar organization showed a well-defined distribution of neuronal and glial populations. In contrast, the analysis of (Cancerous Tissue-P2), shown in Figure 3c,d, revealed significant cytoarchitectural disruption. The regular laminar organization observed in the healthy tissue was entirely replaced by high-grade pleomorphic cellularity.

### 2.2. CellTiter-Glo Assay

The development of increasingly advanced experimental models of human disease, particularly cancer, has historically been a major driving force in drug discovery and therapeutic development [18]. This provided the motivation for establishing physiologically relevant human-derived models, including primary brain cancer cells, to facilitate the discovery and evaluation of novel anticancer therapies. Anticancer drug screening aims to identify natural metabolites that exhibit antitumor activity. Selected compounds are usually toxic, and their anticancer activity is assessed in humans during the clinical phase. The initial screening of cell viability in the brain cancer cell lines U251 and Daoy following treatment with in-house synthesized compounds KCO (49, 50, 51, 54, 60, 62, 64, 65, 69, 70, 73, 126, 128, and 129) was performed using the CellTiter-Glo Luminescent Cell Viability Assay. Cells were exposed to compound concentrations ranging from 50 to 1.5 µM. The standard antineoplastic agents Mitoxantrone and Temozolomide were used as positive controls to induce cell death.

**Figure 4:**
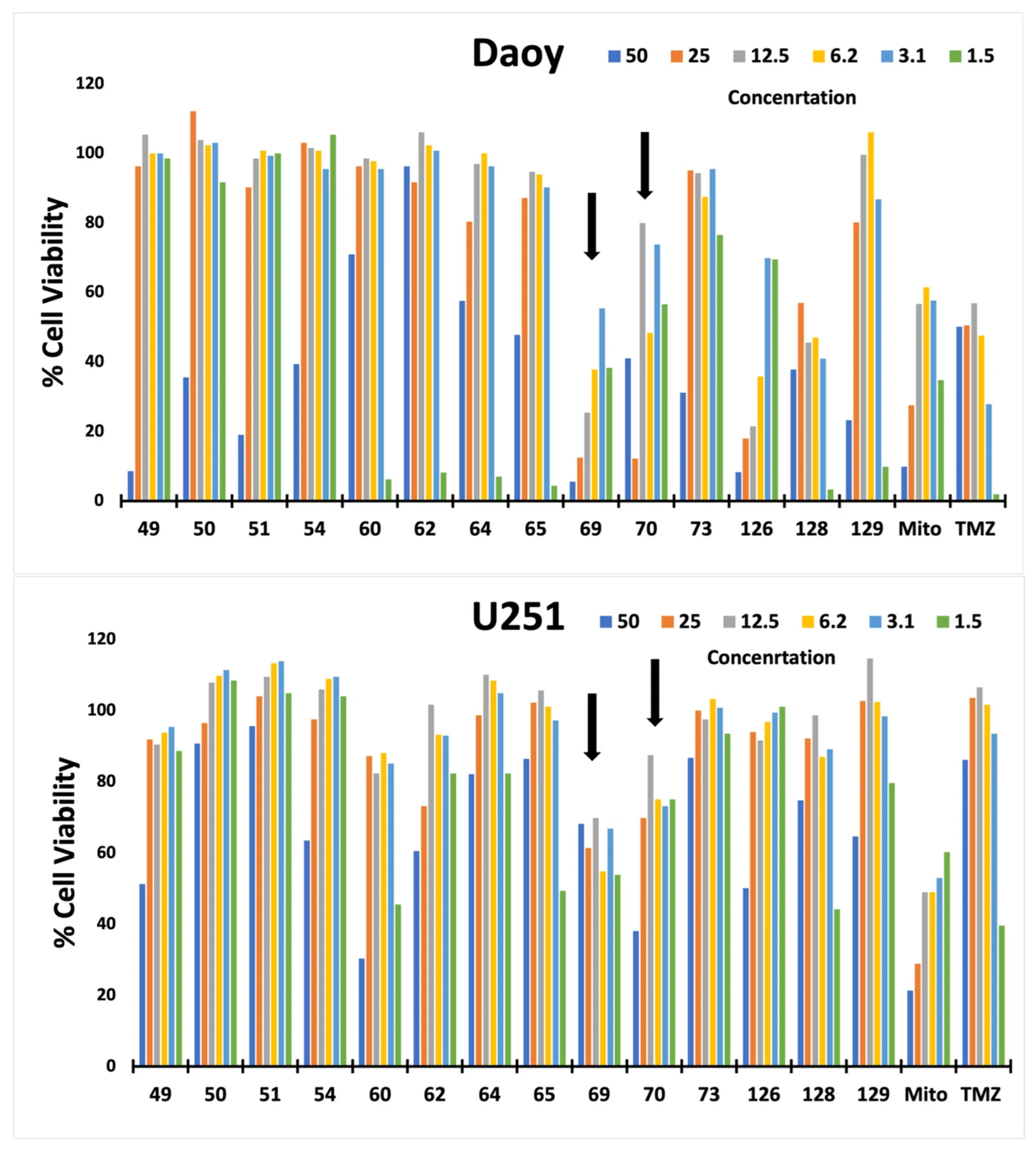
Cell viability screening of synthesized compounds KCO (49, 50, 51, 54, 60, 62, 64, 65, 69, 70, 73, 126, 128, and 129) on U251 and Daoy cell lines. Black arrows show the compounds that were further used in other experiments as lead compounds.

Among the evaluated compounds, KCO69 and KCO70 demonstrated measurable cytotoxic activity, producing a moderate inhibition of tumor cell proliferation compared with the reference drug Mitoxantrone. Notably, these compounds exhibited superior activity compared with most of the tested derivatives. In contrast, the remaining compounds showed minimal or no significant cytotoxic effects under the tested conditions. A summary of the cell viability results obtained from the primary screening assay is presented in Figure 4.

Although compound KCO129 demonstrated promising anticancer activity against breast and colon cancer cell lines in previous studies (unpublished data), it did not produce comparable cytotoxic effects in the brain cancer cell lines (U251 and Daoy) evaluated in the present study. These findings suggest that the compound’s antiproliferative efficacy may be cell-type-dependent, highlighting potential differences in cellular sensitivity or underlying molecular pathways across distinct tumor models.

**Figure 5:**
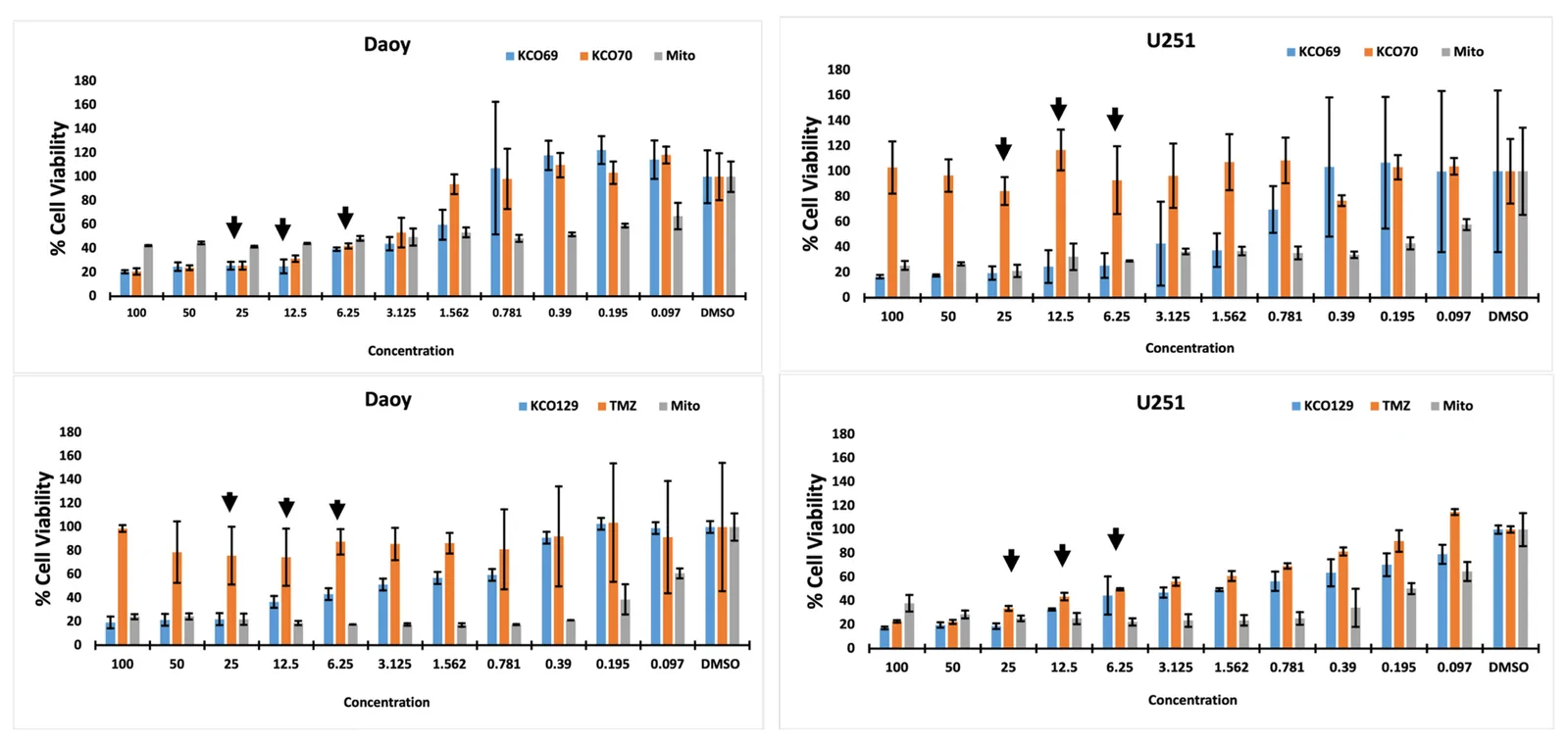
Cell viability screening of serial dilution compounds (KCO69, KCO70, and KCO129) Mito and TMZ on U251 and Daoy cell lines. Black arrow shows the concentrations that we selected and further used in other downstream experiments.

Based on the results presented in Figure 5, the concentrations 25, 12.5, and 6.25 μM were selected for subsequent evaluation in patient-derived primary cell lines. These concentrations were chosen to further investigate the cytotoxic effects of the tested compounds and to assess their activity in a more clinically relevant cellular model.

The antiproliferative activity of the tested compounds was evaluated using the CellTiter-Glo Luminescent Cell Viability Assay in human brain tumor-derived primary cells (P2, 3, 5, 8, 12, and 13), Normal Tissue (P6), and the immortalized cell lines U251 and Daoy. Cells were exposed to varying concentrations of compounds KCO69, KCO70, and KCO129 (25, 12.5, and 6.25 μM), and cell viability was quantified by measuring intracellular ATP levels, which serve as an indicator of metabolically active cells.

Treatment with all three compounds resulted in a dose-dependent reduction in cell viability compared with untreated control cells. The decrease in viability became more pronounced at higher concentrations, particularly at 25 µM and 12.5 µM, indicating a strong cytotoxic effect at these doses.

Among the tested compounds, KCO70 demonstrated the most potent antiproliferative activity, producing a greater reduction in cell viability relative to KCO69 and KCO129 at equivalent concentrations. These findings suggest that KCO70 may exert stronger cytotoxic effects on human tumor-derived cells.

**Figure 6:**
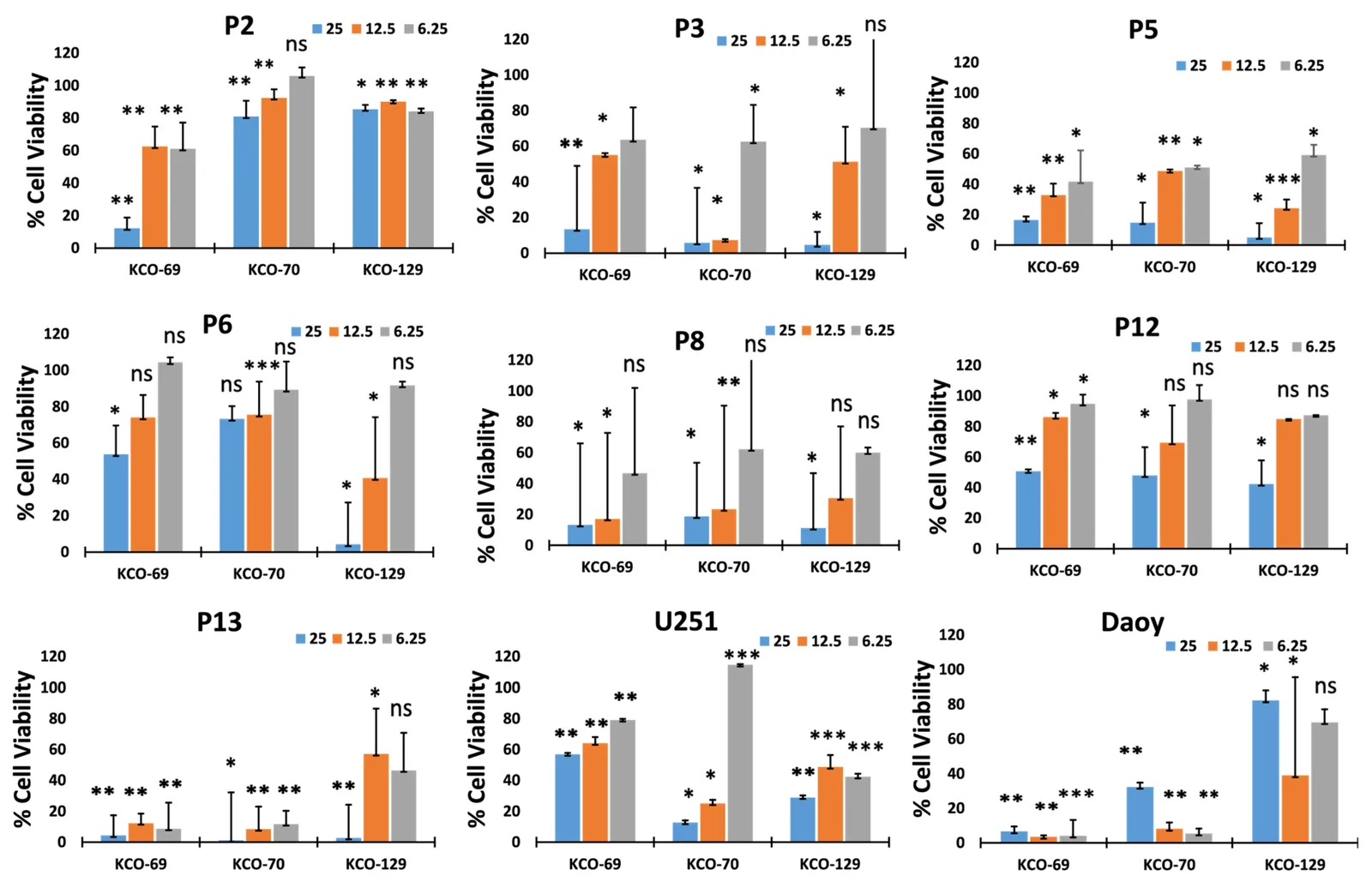
Treatment of patient explant-derived cells with KCO69, KCO70, and KCO129. Cell viability was assessed by Titer Glo assay. Among all samples, P3, P5, and P13 showed more than 50% killing even at 12.5 µM, compared with Normal Tissue (P6), suggesting the promise of these compounds for brain tumor research *p*-value of <0.05. *p*-values ≥ 0.1 = ns, 0.05–0.02 = *, 0.01–0.002 = ** and ≤0.001 = ***.

Overall, the results indicate that compounds KCO69, KCO70, and KCO129 possess notable anticancer potential, with KCO70 showing comparatively enhanced activity. This compound may therefore represent a promising candidate for prioritization in subsequent mechanistic studies, and further preclinical evaluation results showcased in Figure 6 and IC_50_ are shown in Table 1.

The inclusion of normal primary human cells in anticancer drug discovery is essential for evaluating drug selectivity, minimizing cytotoxic effects on healthy tissues, and improving the translational relevance of preclinical findings [19,20]. And based on that, it was isolation and growth of normal primary brain cell from patient 6 (P6), diagnosed with refractory seizure and presenting cerebral tissue characterized by gliosis and remote hemorrhage consistent with trauma-related changes, was used as a non-cancerous control.

Comparative analysis between the control (Normal Tissue, P6) and human brain tumor-derived primary cells (P2, P3, P5, P8, P12, and P13) demonstrated that the control cells exhibited greater resistance to the tested compounds (KCO69, KCO70, and KCO129).

**Figure 7:**
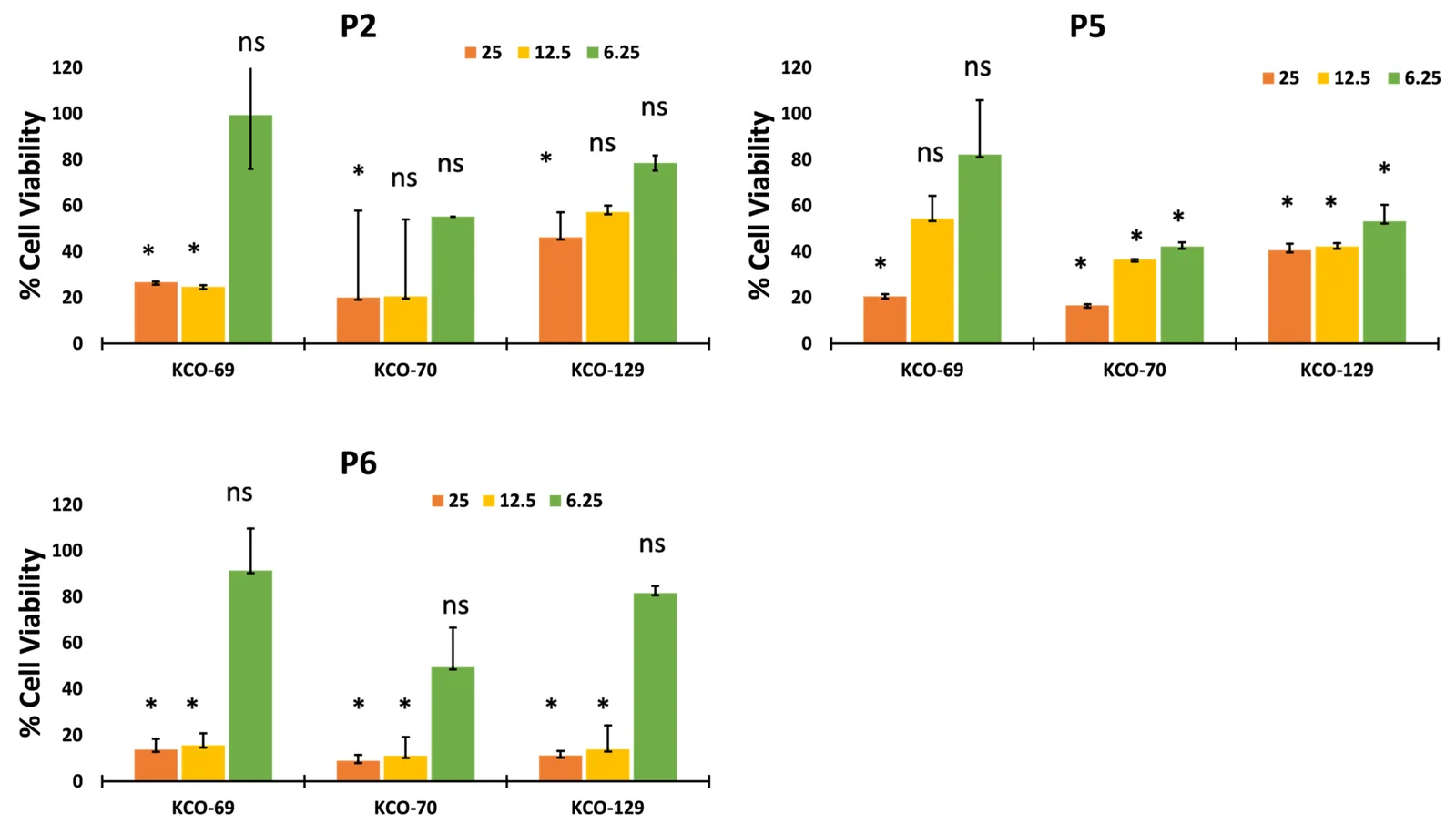
Treatment of Primary Brain cell (P2, P5, U251, Daoy) and Normal Tissue (P6, Control) with Novel compounds (KCO69, KCO70, KCO129). Cell viability was assessed by MTT assay *p*-value of <0.05. *p*-values ≥ 0.1 = ns, 0.05–0.02 = *.

These findings suggest that the evaluated compounds display selective cytotoxicity toward tumor cells, with minimal effects on Normal Tissue, as clearly supported by both MTT and Titer-Glo assay results, as shown in Figure 6 and Figure 7.

**Figure 8:**
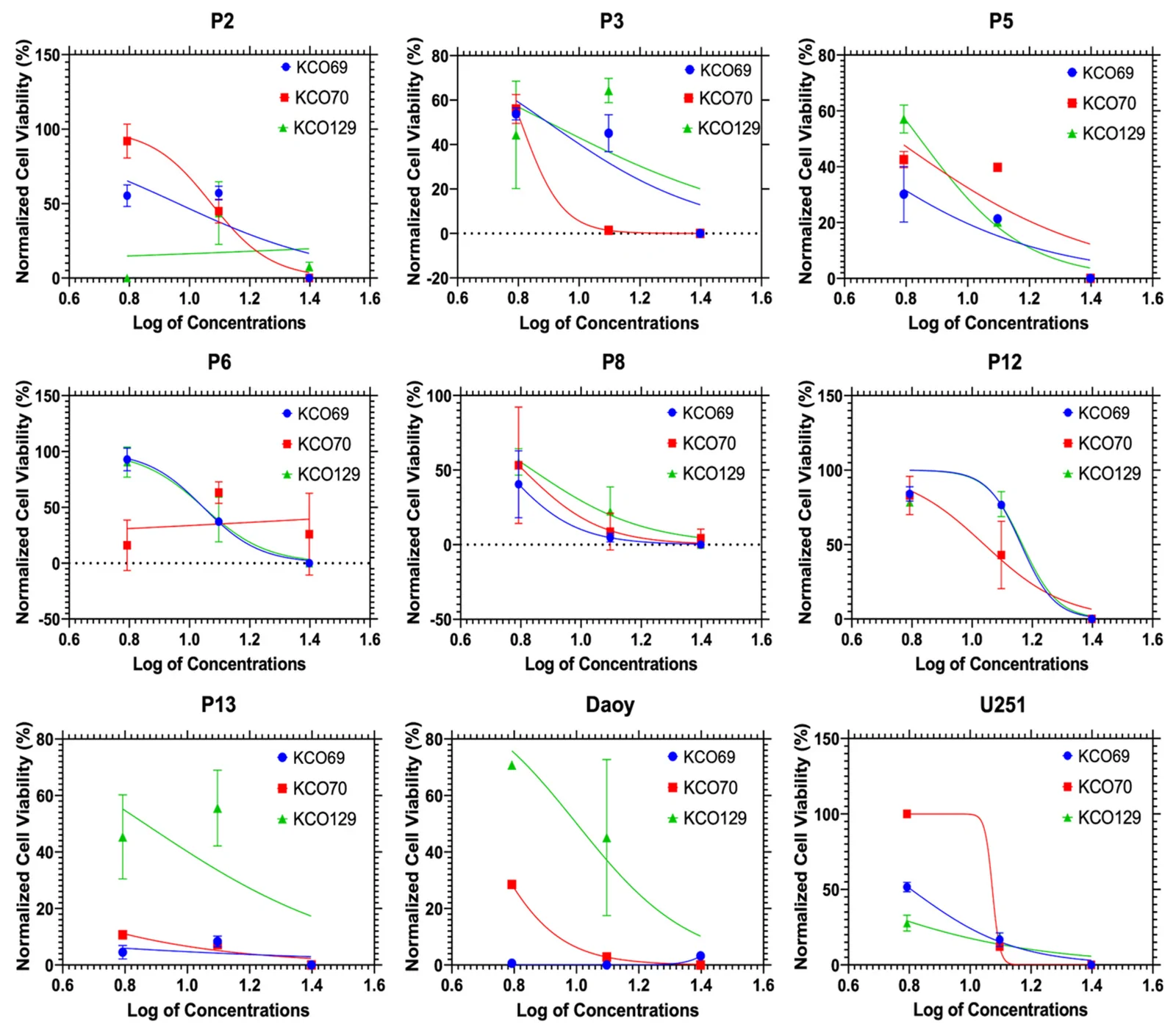
Cell viability curves of the selected compounds (KCO69, KCO70, KCO129) against the patient’s tumor-derived cell lines and cancer cell lines (U251, Daoy).

Cell viability curves of the selected compounds against the patient’s tumor-derived cell lines are shown in Figure 8. Compound KCO70 exhibits superior activity compared to other tested compounds against the P2, P8, P12 and U251 cancer cell lines. Whereas compound KCO69 exhibited an excellent activity profile against P12 and P13 compared to the U251 cell line.

### 2.3. Cellular Metabolic Activity Assays

Cell-based compound profiling and drug dose–response assays, including MTT and CellTiter-Glo assays, play a critical role in both fundamental biomedical research and anticancer drug discovery by enabling the quantitative evaluation of cellular viability, proliferation, and therapeutic response following compound treatment. The cytotoxic effects of the compounds KCO69, KCO70, and KCO129 were evaluated using MTT and CellTiter-Glo assays against human tumor-derived cell lines cultured in 96-well plates. Cells were treated with varying concentrations of each compound (25, 12.5, and 6.25 μM) and incubated for 48 h at 37 °C in a humidified atmosphere containing 5% CO_2_. The half-maximal inhibitory concentration (IC_50_) was determined for each compound across the tested concentrations. Cytotoxic activity was quantified using the MTT assay by calculating the mean % cell viability. All synthesized compounds exhibited concentration-dependent cytotoxic effects against patients’ cancer cell lines, with notable anticancer activity at an IC_50_ of 12.5 μM (Figure 7).

Based on the obtained results, compound KCO70 demonstrated comparatively greater anticancer activity than compounds KCO69 and KCO129. Pathway enrichment analysis was subsequently performed to explore the potential molecular mechanisms underlying the observed biological activity.

### 2.4. In Silico Analysis

As summarized in Table 2, analysis use pathway databases such as the KEGG Pathway Database and the Reactome Pathway Database. Cellular life is governed by complex networks of molecular reactions, including signal transduction, transport, DNA replication, protein synthesis, and metabolism. Several databases, such as Rhea, KEGG, MetaCyc, and PANTHER, document these processes at the levels of individual reactions or pathways. The Reactome Knowledgebase uniquely focuses on *Homo sapiens*, integrating these reactions into a unified, systematically annotated network that links proteins to their molecular functions and supports the discovery of novel biological relationships [21]). The Reactome Pathway Database suggested that compound KCO70 may be associated with several cancer-related biological pathways. Among the most significant pathways identified were Polycyclic Aromatic Hydrocarbon Degradation (*p*-value = 0.0209) and Constitutive Signaling by Aberrant PI3K in Cancer (*p*-value = 0.0289).

These findings suggest that the enhanced anticancer activity observed for compound KCO70 may be mediated by modulation of metabolic detoxification processes and interference with oncogenic signaling pathways, particularly those involving aberrant PI3K activation. Collectively, these pathway associations provide insight into the potential mechanisms by which compound KCO70 may exert its anticancer effects, supporting its prioritization for further mechanistic and functional investigations.

Our compounds feature a heavily conjugated system terminating in a highly electrophilic/acceptor Meldrum’s acid alkylidene core alongside a traditional kinase-inhibiting quinoline core. It is highly likely to act via one of two distinct mechanisms: Kinase Inhibition: Quinoline structures frequently target ATP-binding pockets of tyrosine kinases such as EGFR, VEGFR, or MET. Michael Acceptance/Covalent Inhibition: The exocyclic double bond on the Meldrum’s acid ring is highly electron-deficient and can act as a Michael acceptor, potentially targeting soft nucleophiles (like cysteine residues) in cancer-related enzymes.

We have performed target prediction using SwissTarget Prediction (Table 3 and Supplementary Figure S1). This is useful for understanding the molecular mechanisms underlying a given phenotype or bioactivity, rationalizing potential favorable or unfavorable side effects, predicting off-targets of known molecules, and clearing the way for drug repurposing. Predictions are made using a ligand-based approach that assesses similarity between a query molecule and known ligands from a large collection of protein targets. The SwissTargetPrediction tool (https://www.swisstargetprediction.ch/, accessed on 24 May 2026) uses dual scoring of molecular similarity based on 2D and 3D approaches and allows predictions across several species. Supplementary Figure S1 shows the target classes of the three compounds. The family AG protein-coupled receptors and ligand-gated ion channels account for 33.3% of the target class in KCO69 and KCO70, respectively, whereas compound KCO129 showed greater diversity, with 20% of each being ligand-gated ion channels, transferases, and erasers. Interestingly, all three compounds have ligand-gated ion channels in common as a target class.

This firmly establishes why this specific conjugated framework possesses target-specific antiproliferative properties, differentiating it from simple, unstructured quinoline molecules.

The patient-derived primary brain cancer cultures (P3, P5, P8, and P13) exhibited heterogeneous responses to treatment with the tested compounds. Among these samples, P8 and P3 showed the most pronounced reduction in cell viability, indicating greater sensitivity to the compounds than the other primary cultures.

Clinical diagnosis indicated that the P8 sample corresponded to glioblastoma, a malignancy commonly associated with multiple genetic alterations, including mutations in EGFR, TERT, and TP53, which contribute to abnormal cell proliferation and genomic instability. In addition, the P3 was diagnosed with neurofibromatosis type 2, a condition caused by mutations in the NF2 gene that regulates cell growth and tumor suppression.

The pronounced sensitivity observed in the P3 and P8 cultures suggests that compounds KCO69 and KCO70 may exert stronger anticancer effects in tumors harboring these specific molecular alterations. This observation suggests a potential association between these compounds’ activity and tumor-related signaling pathways, supporting the hypothesis that their therapeutic efficacy may be influenced by the tumor’s underlying genetic and molecular characteristics.

### 2.5. Immunocytochemistry (ICC)

**Figure 9:**
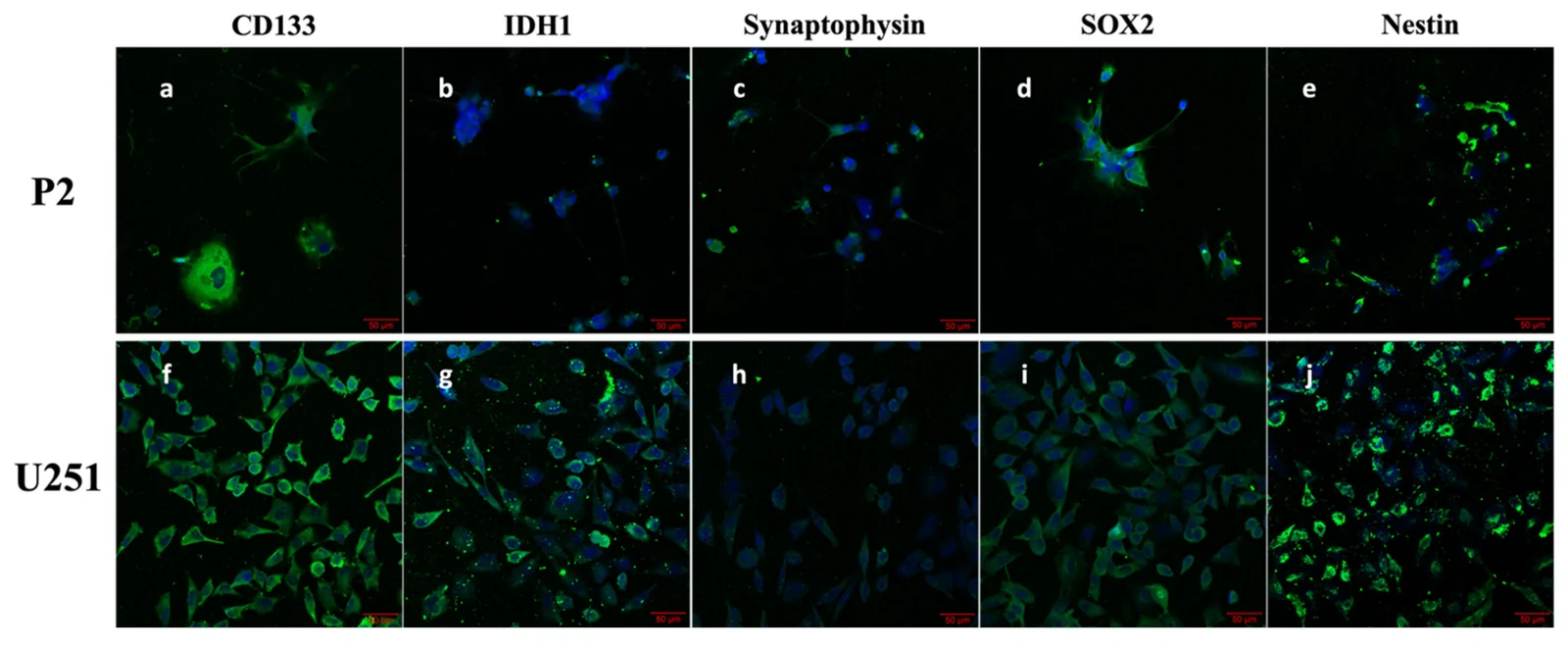
Immunocytochemistry images of a panel of markers to perform a comprehensive profiling of the patient-derived explanted cells and a brain cancer cell line. Patient-derived cells (P2) and brain cancer cell line U251 were stained for (**a**,**f**) CD133, (**b**,**g**) IDH1, (**c**,**h**) Synaptophysin (**d**,**i**) SOX2 and (**e**,**j**) Nestin. Green color shows the protein of interest, and blue color depicts the Nucleus staining. Scale bars = 50 μm.

Immunocytochemistry (ICC) analysis was performed using a panel of biomarkers, including Synaptophysin, SOX2, CD133, and IDH1, due to their well-established relevance in primary astrocytoma and glioblastoma brain tumor cell characterization. These markers were selected to assess neural differentiation, stem cell-like properties, and tumor-associated phenotypic features in the isolated primary brain cancer cells [23,24]. To confirm that the explanted samples are true representatives of the primary brain tumor-derived cell population, the samples were subjected to immunocytochemistry (ICC) to evaluate the expression of selected neural and tumor-associated markers. Representative images from sample P2 and U251 cell line are presented in Figure 9. The analysis revealed a remarkable expression of the antigens CD133 shown in Figure 9a,f, IDH1 Figure 9b,g, and Nestin Figure 9e,j in P2 cells and U251 cell line. The strong immunoreactivity of these markers is consistent with characteristics commonly associated with neural progenitor and brain tumor-derived cells. In addition, slight expression of Synaptophysin shown in Figure 9c,h and SOX2 Figure 9d,i was also observed. Although the staining intensity for these markers was relatively low, their presence further supports the neural lineage and tumor-associated phenotypes of the cultured cells.

Collectively, the ICC findings confirm that the P2 cell population retains key molecular markers associated with primary brain tumor cells, supporting its suitability as an in vitro model for subsequent functional and therapeutic investigations, as shown in Figure 9.

## 3. Materials and Methods

### 3.1. General Precautions

Human tissue samples may contain infectious agents; therefore, stringent precautions were observed throughout all stages of sample handling and processing. Personnel involved in the processing of human tissue were required to complete relevant training and adhere strictly to the institutional Environmental Health and Safety (EHS) guidelines and protocols.

1. All activities were performed in a physical containment 2 (PC2) laboratory, in a dedicated Class II B2 biological safety bio cabinet.
2. Strict aseptic techniques were employed under the following conditions:
  (a) Prior to use, the work surface of the biological safety cabinet was thoroughly wiped with 80% (*v*/*v*) ethanol. All materials and equipment were sprayed with 80% ethanol before being introduced into the cabinet.
  (b) Biological waste was disposed of in a sealed container containing a 10% bleach solution within the biological safety cabinet to minimize the risk of aerosol exposure.
  (c) Personnel wore a double layer of nitrile gloves during tissue processing, and care was taken to prevent skin punctures. The outer layer of gloves was discarded into a designated hazardous waste bag before the hands were withdrawn from the biological safety cabinet.

### 3.2. The Source of the Tissue

#### Clinical History

Tissue samples were obtained after patient informed consent, as approved by the institutional ethics committee of KAIMRC. Table 4 lists all samples collected during this study.

### 3.3. Experimental Design

#### 3.3.1. Sample Collection (Brain Tissue)

**Figure 10:**
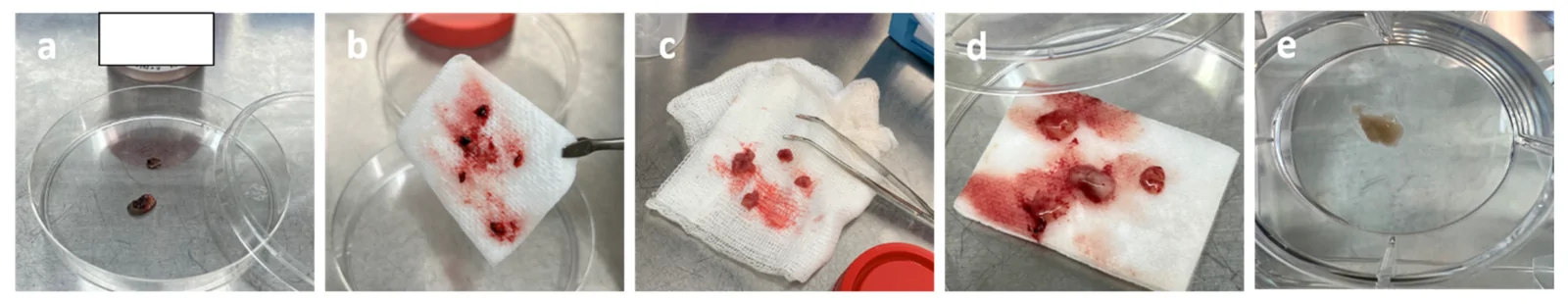
(**a**–**e**) Brain tissue samples were obtained in a range of sizes. Size ranges from a few cm to a few mm.

A sample of brain tissue collected during various neurosurgical procedures can therefore be a valuable source for isolating neurons, astrocytes, oligodendroglia, microglia, ependymal and microvascular cells, and various neoplastic cells from brain tumors. We collected brain tissue samples after the surgery, which were obtained in a range of sizes (Figure 10a–e).

**Figure 11:**
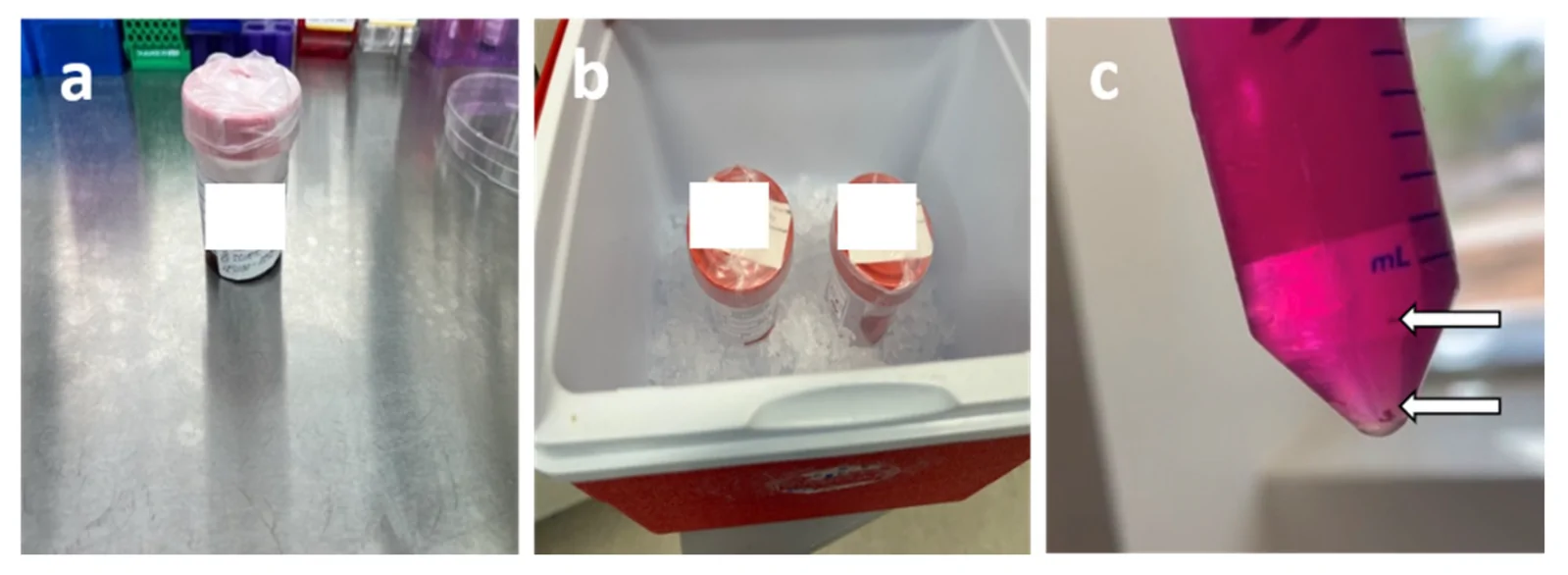
(**a**) Brain tissue specimen prepared in media for transport to the laboratory. (**b**) Samples were kept on ice for transportation in a thermostable box. (**c**) Small pieces of brain specimen in a Falcon tube ares ready for transportation (White arrows).

Brain tissue is collected under sterile conditions from cortical and subcortical regions, depending on the surgery performed. When feasible, both gray matter and underlying white matter are removed during surgery. After surgical resection, fragments of viable tissue are collected, placed in cell culture medium or saline to prevent desiccation, and immediately transported to the laboratory (Figure 11).

#### 3.3.2. Preparation of Tissue for Cell Culture

The protocol below describes the steps required to process a whole-tumor tissue specimen into a homogenized cell suspension for tumor cell isolation.

**Figure 12:**
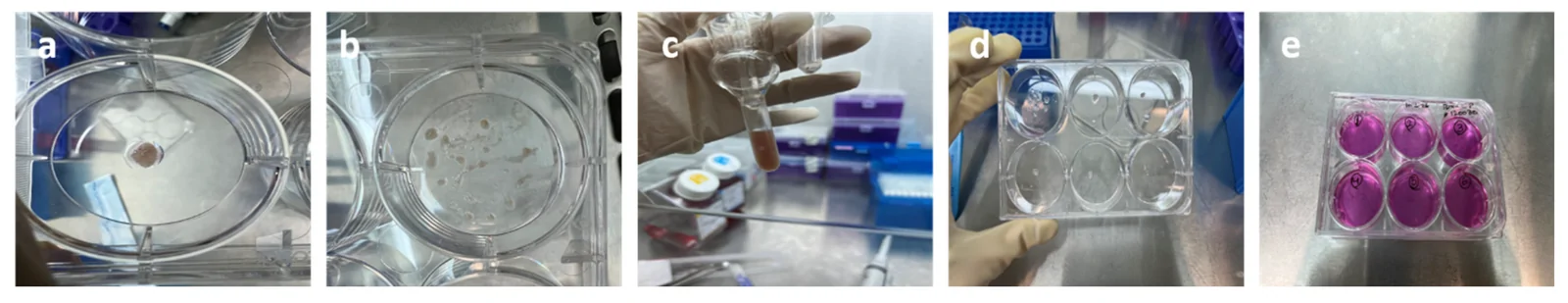
Steps of preparation of tissue for cell culture: (**a**) tissue washing, (**b**) tissue cutting, (**c**) dissociation manual by digestion tools, (**d**) seeding into a 6-well culture plate, (**e**) explants were cultured in different media conditions to optimize viability.

- Tissue fragments were washed in Hank’s Balanced Salt Solution (HBSS) (Figure 12a).
- After centrifugation, the supernatant was carefully discarded by manual aspiration, taking care not to dislodge the specimen.
- The sample was transferred to a sterile 10 cm tissue culture dish pre-cooled on ice.

Note: Ensure the tissue does not dry out in subsequent steps; add PBS as needed.

- Working quickly, a scalpel blade was used to finely mince the tissue and cut it into small pieces to achieve coarse mechanical decomposition (Figure 12b).
- Once the tissue is finely minced, it is collected into a 50 mL Falcon tube:
  - The minced tissue was transferred by tilting the dish at an angle and rinsing the dish with 10 mL of PBS with a 25 mL serological pipette.
  - This process was repeated until all tissue had been collected into the conical tube.
  - The sample was centrifuged at 300× *g* for 5 min at 4 °C.

Note: Pre-wet all serological pipettes and filtered pipette tips with PBS just before use to prevent tissue specimens from lodging on the walls of the plasticware.

- Then the tissue sample was incubated for 1–2 h with enzyme dissociation medium (1 mL of HBSS + 1 μL dispase1 + 0.1 μL DNase 1 + 2.5 μL collagenase) with manual flicking every 15 min to allow more cells to dissociate.
- Additionally, to enhance the digestion process, the tissue was processed with manual digestion tools (Figure 12c).
- This is followed by centrifugation at 300× *g* for 3 min.
- Discard the supernatant after centrifugation by carefully but swiftly decanting directly into the waste container without disturbing the pellet.
- Using a 10 mL serological pipette, 3 mL of DMEM media was added, and the cell pellet was triturated until no visible large pieces were present. If large pieces remain, repeat enzymatic dissociation.
- This is followed by centrifugation at 300× *g* for 3 min.
- The sediment is resuspended again in cell culture medium-containing antibiotics Pen/Strep 5% and fetal bovine serum (FBS) 10%, and plated out into a 6-well culture plate (Figure 12d).
- The resulting cell suspension was incubated at 37 °C and 5% CO_2_ for approximately one month, resulting in preferential proliferation and survival of brain cells. The medium is normally changed twice a week. Explants were cultured in different media conditions to optimize viability (Figure 12e).
- Cell culture media used for primary cells and their recipe (Table 5).

**Figure 13:**
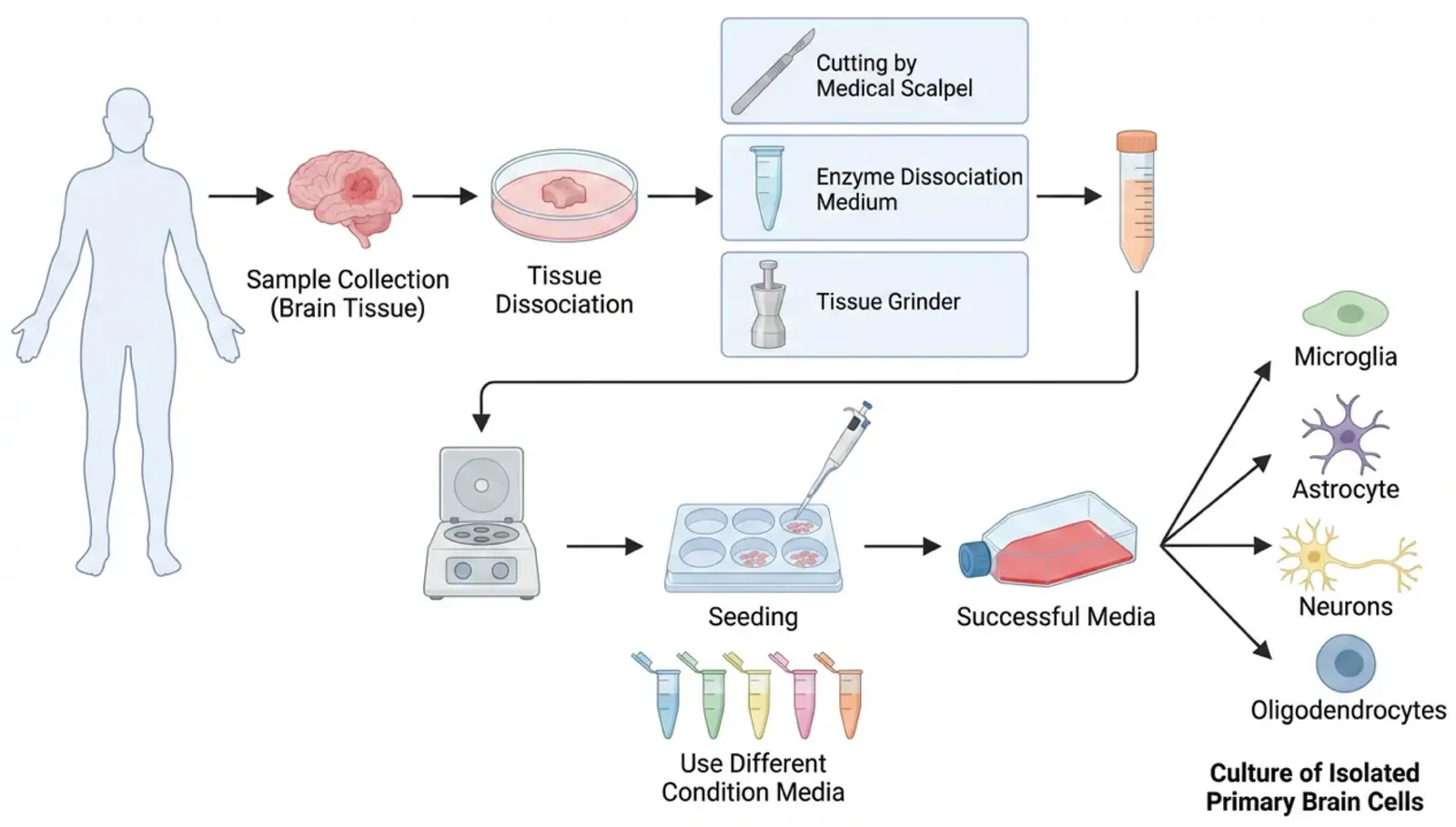
Flow chart of the isolated primary brain cells from human tissue.

Figure 13 illustrates the experimental workflow, beginning with the initial acquisition of cancer samples, followed by either enzymatic digestion or direct plating of tissue specimens. Subsequently, cells were cultured in various culture media to optimize growth conditions, ultimately establishing diverse brain cell populations maintained in vitro.

#### 3.3.3. The Culture of Isolated Primary Brain Cells

**Figure 14:**
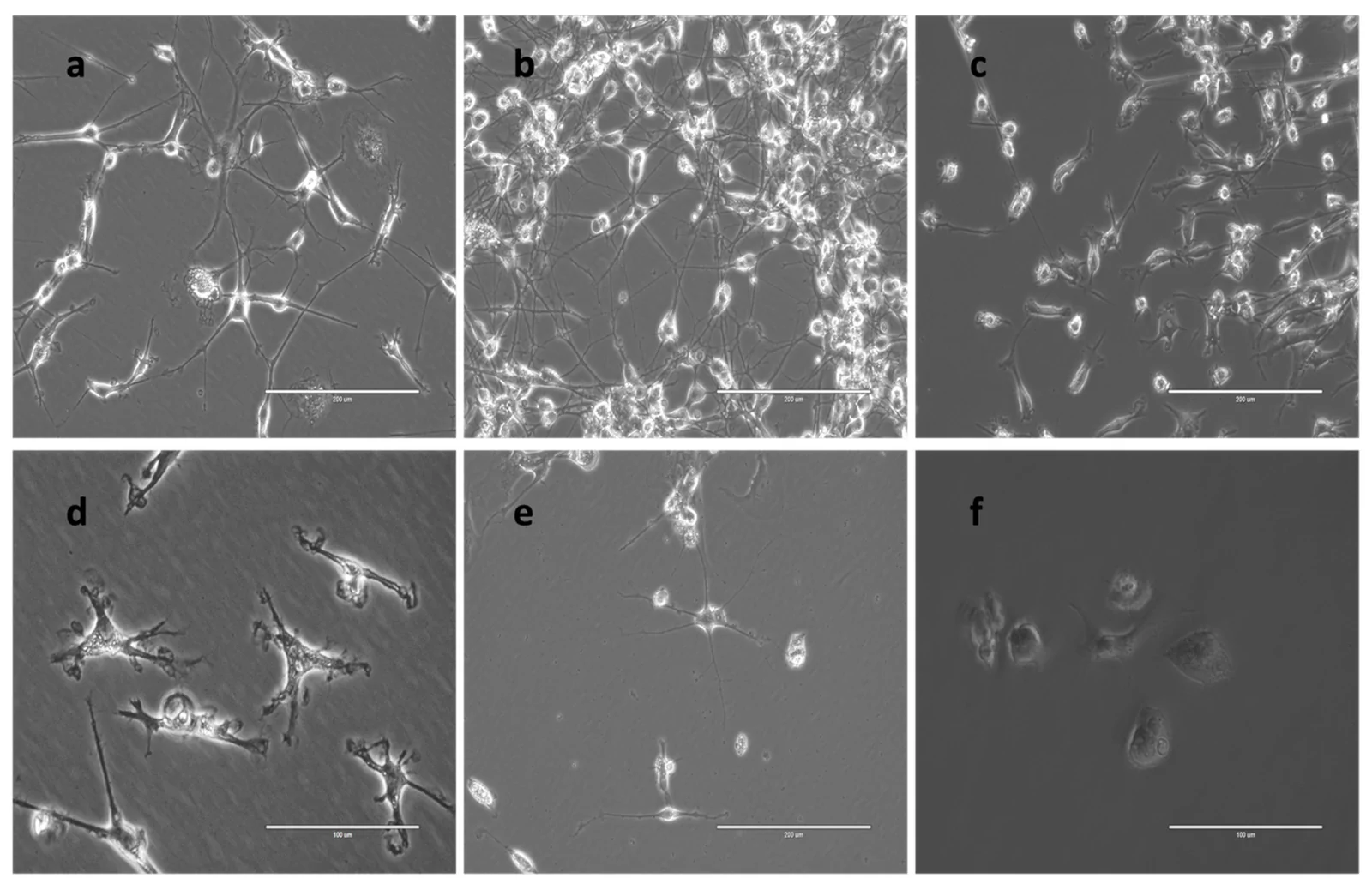
Patient-derived primary brain cells (**a**) show primary multipolar neuron cells. Scale bar = 200 µm (P2). (**b**) Network of neurons, scale bar = 400 µm (P2). (**c**) Shows primary neuron cells, scale bar = 200 µm (P5). (**d**) Primary microglia, scale bar = 200 µm (P3). (**e**) Primary astrocytes (P6). (**f**) Shows neural stem cells (NSCs) (P6), scale bar = 100 µm.

Continuous culture of tissue samples resulted in neuron cells adhering to the culture ware, and outgrowth of cells attached to the tissue on the cell culture plate was observed. The outgrowth of large primary tumor neuron-like cells (Figure 14a,f) was visible within a few days, then the primary neuron cells were cultured in culture flasks and incubated at 37 °C in a controlled atmosphere with 5% CO_2_.

### 3.4. Cytology Analysis (Characterization)

#### 3.4.1. Cell Viability and Proliferation

Cell proliferation and cytotoxicity assays constitute fundamental experimental approaches for characterizing the cellular effects of drug candidates, enabling the systematic evaluation of their therapeutic efficacy and safety during the drug discovery and development pipeline [25,26]. In this study, cytotoxicity was assessed using the MTT and CellTiter-Glo assays.

#### 3.4.2. Cell Culture and Treatment

Cells isolated from primary brain samples Normal Tissue (P6), Cancer Tissue (P2, P5), the human malignant glioblastoma multiforme (GBM) U251 (09063001, Merck, Darmstadt, Germany), and the human medulloblastoma Daoy-HTB-186 (ATCC) cell lines were exposed to various concentrations of locally synthesized novel anthracene-based compounds (KCO69, KCO70, KCO129) and Temozolomide. The U251 cell line served as a control. These cells were treated with different concentrations of KCO69, KCO70, and KCO129 along with TMZ, an anticancer drug used to treat brain cancer.

Cells were seeded at a density of 1 × 10^4^ cells per well in 96-well plates and incubated overnight at 37 °C in a humidified atmosphere containing 5% CO_2_ to allow for cell attachment. Subsequently, cells were treated with the test compounds and TMZ using serial dilutions ranging from 100 µM to 0.097 µM, followed by a 48 h incubation period. For the MTT assay, a stock solution of MTT (5 mg/mL) was prepared in distilled water and diluted 1:1000 in fresh culture medium prior to use. A 5 µL aliquot of the diluted MTT solution was added to each well, and the plates were incubated for 2–4 h to allow formation of formazan crystals. After incubation, the culture medium was carefully removed without disturbing the cell monolayer, and dimethyl sulfoxide (DMSO) was added to solubilize the formazan crystals. Plates were wrapped to protect from light and placed on an orbital shaker for 30–45 min until complete dissolution was achieved. Absorbance was measured using a SpectraMax Plus 384 spectrophotometers (Molecular Devices, San Jose, CA, USA) at 560 nm, with background correction at 650 nm. The absorbance values were normalized and expressed as relative percentages of cell viability. Data analysis was performed using GraphPad Prism 8 software, and the half-maximal inhibitory concentration (IC_50_) values were calculated accordingly.

#### 3.4.3. The CellTiter-Glo Assay

Quantification of adenosine 5′-triphosphate (ATP), commonly referred to as the ATP assay, is a widely used method for assessing cell viability by measuring total intracellular ATP levels. This assay is based on a bioluminescent detection principle [25].

#### 3.4.4. Cell Culture and Treatment

Primary Cells (P2, P3, P5, P6, P8, P12, and P13) were seeded in white 96-well plates at a density of 1 × 10^4^ cells per well and incubated overnight at 37 °C with 5% CO_2_. The cells were then treated with varying concentrations of each compound (25, 12.5, and 6.25 μM) for 48 h. Cell viability was assessed using the CellTiter-Glo 2.0 assay (Promega, Madison, WI, USA), by adding 50 µL of CellTiter-Glo 2.0 Reagent to each well. The plate was wrapped and placed on an orbital shaker for 2 h to induce cell lysis. Luminescence readings were recorded using the Perkin Elmer Multimodal Plate Reader, Envision system.

#### 3.4.5. Immunocytochemistry (ICC)

Cells (P2) were prepared by seeding 1 × 10^4^ cells per well into sterile black 96-well plates using complete growth medium, followed by incubation at 37 °C in a humidified atmosphere containing 5% CO_2_. For fixation, the culture medium was aspirated, and cells were gently washed with phosphate-buffered saline (PBS) at room temperature. Cells were then fixed with 100 µL of a solution containing 10% formalin and 4% formaldehyde for 1 h at room temperature. The fixative was carefully removed, and cells were washed with PBS for 2 min. To minimize background fluorescence, cells were blocked with 50 µL of 1% bovine serum albumin (BSA) for 1 h at room temperature. The blocking solution was discarded, and cells were washed twice with PBS prior to incubation with the primary antibody (1:100 dilution) overnight at 4 °C. In this study we used CD133 (Mouse, Invitrogen, Carlsbad, CA, USA, 5E3), IDH1 (Mouse, Invitrogen, GT1521), Synaptophysin (Rabbit, Invitrogen, AP11), Nestin (Mouse, Invitrogen, 10C2) and Sox2 (Rabbit, Invitrogen, SP76) antibodies. After removal of the primary antibody, cells were washed three times with PBS for 5 min each and subsequently incubated with the appropriate secondary antibody (raised in the same species as the blocking serum) for 1 h at 4 °C in the dark. Following secondary antibody incubation, cells were washed three times with PBS for 5 min each. For nuclear counterstaining, cells were incubated with Hoechst 33342 (1:2000 dilution; 50 µL per well) for 5 min at room temperature, followed by washing with PBS. Fluorescence imaging was performed using a confocal laser scanning microscope Zeiss cLSM780 (Zeiss Oberkochen, Germany), and images were captured under appropriate excitation and emission settings.

### 3.5. Statistical Analyses

Student’s *t*-test or one-way analysis of variance (ANOVA) was used to compare two or more groups, respectively. Tails specify the number of distribution tails to return: paired, one-tailed distribution was used =1.

Statistical significance was set at a *p*-value of <0.05. *p*-values ≥ 0.1 = NS, 0.05–0.02 = *, 0.01–0.002 = ** and ≤0.001 = ***.

## 4. Conclusions

The complexity and heterogeneity of cancer have posed significant challenges to the global scientific community for decades. A comprehensive understanding of the intricate molecular and cellular landscape of cancer remains a central objective in cancer biology. Immortalized cancer cell lines serve as invaluable models for studying tumor biology, offering insights into the genomic, proteomic, and molecular features of various cancer types. However, identifying an appropriate cell line that accurately recapitulates the biological behavior of primary tumors remains a persistent challenge. Furthermore, extensively passaged cell lines often exhibit altered morphology, gene expression patterns, and proteomic profiles, which can compromise the validity of experimental findings. Therefore, establishing explanted cells derived directly from primary tumors—preferably at low passage numbers and exhibiting distinct cancer-specific characteristics—is essential for advancing translational cancer research.

In conclusion, the successful isolation, expansion, and comprehensive characterization of Saudi patient–derived brain cancer cells establish a robust, biologically relevant, and clinically reflective in vitro platform for preclinical drug discovery. These models preserved key histopathological and molecular characteristics of the original tumors, including stemness and tumor-associated markers, while maintaining interpatient heterogeneity—an essential feature often lacking in conventional immortalized cell lines.

The demonstrated cytotoxic activity of novel compounds KCO69, KCO70, and KCO129, particularly at higher concentrations, highlights their promising therapeutic potential. The concentration-dependent reduction in cell viability observed through both MTT and ATP-based (Titer-Glo) assays further strengthens the reliability of the findings and confirms metabolic and proliferative suppression in treated cells. Among the tested candidates, compound KCO70 exhibited comparatively stronger antiproliferative effects, suggesting potential for prioritization in subsequent investigations.

Importantly, the variability in drug responsiveness across different patient-derived cultures underscores the translational relevance of these models and reflects the molecular and phenotypic diversity characteristic of brain tumors. Collectively, these findings support further mechanistic studies, dose-optimization analyses, and in vivo validation to explore the therapeutic applicability of the investigated compounds. Moreover, this work reinforces the critical value of patient-derived primary models in advancing precision oncology approaches and developing population-specific therapeutic strategies for brain cancer management in Saudi Arabia and beyond.

## Figures and Tables

**Table 1:** Anticancer activity of most active compounds against various cell lines (IC_50_ in μM).

**KCO**	**U251**		**Daoy**		**P2**		**P3**		**P5**	
	**IC_50_**	**R^2^**	**IC_50_**	**R^2^**	**IC_50_**	**R^2^**	**IC_50_**	**R^2^**	**IC_50_**	**R^2^**
69	6	0.98	35	0.92	9	0.67	7	0.75	3	0.7
70	11	1.00	4	0.99	11	0.97	6	0.98	5	0.67
129	3	0.8	10	0.8	7	0.01	7	0.33	6	0.98
**KCO**	**P6**		**P8**		**P12**		**P13**			
	**IC_50_**	**R^2^**	**IC_50_**	**R^2^**	**IC_50_**	**R^2^**	**IC_50_**	**R^2^**		
69	11	0.98	5	0.79	14	0.93	0.03	0.17		
70	12	0.01	6	0.6	10	0.89	1.11	0.8		
129	11	0.93	6	0.88	14	0.87	7	0.4		

**Table 2:** Data generated using the iFragMent server. It assigns chemical compounds to biological pathways using a profile-based approach [22].

Compound	Structure	Molecular Pathways	Database	Confidence (*p*-Value)
KCO69	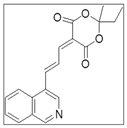	Polycyclic aromatic hydrocarbon degradation	KEGG Pathways	0.0251
		Type I polyketide structures		0.0362
		Constitutive Signaling by Aberrant PI3K in Cancer	Reactome	0.0319
KCO70	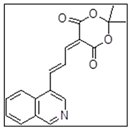	Polycyclic aromatic hydrocarbon degradation	KEGG Pathways	0.0209
		Constitutive Signaling by Aberrant PI3K in Cancer	Reactome	0.0289
KCO129	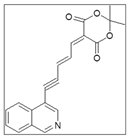	Biosynthesis of enediyne antibiotics	KEGG Pathways	0.0225
		Constitutive Signaling by Aberrant PI3K in Cancer	Reactome	0.0587

**Table 3:** Data generated using the SwissDock server.

Compound	Structure	Molecular Target Prediction	Target Class
KCO69	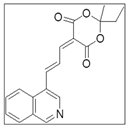	Amyloid-beta precursor protein (APP)	Membrane Receptor
		Protein kinase C alpha type (PRKCA)	Kinase
KCO70	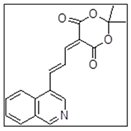	Amyloid-beta precursor protein (APP)	Membrane Receptor
		NAD-dependent protein deacetylase sirtuin-1 (SIRT1)	Eraser
KCO129	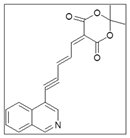	Amyloid-beta precursor protein (APP)	Membrane Receptor
		Poly [ADP-ribose] polymerase 1 (PARP1)	Transferase

**Table 4:** Cases of brain cancer patients participated in the research. M = Male and F = Female.

Abbreviation	Diagnosis	Age	Gander	Location
P1	Brain Tumor—Metastatic Carcinoma	68	M	Right frontal
P2	Brain Tumor—High-Grade Astrocytoma, WHO grade 4	33	M	Left frontal
P3	Brain Tumor—Schwannoma, WHO grade 1	43	M	Right sciatic nerve tumor
P4	Meningioma	48	F	Olfactory groove
P5	Brain Tumor—Astrocytoma, WHO grade 2	38	M	Right parietal lobe
P6	Cerebral Tissue Control	24	M	Right frontal
P7	Mammosomatotroph PitNET/Adenoma	39	M	Pituitary region
P8	Brain Tumor—Glioblastoma, WHO grade 4	39	M	Left temporal
P9	Brain TumorI—nfant-type Hemispheric Glioma	9 M	F	Right temporoparietal
P10	Metastatic Colonic Adenocarcinoma	61	M	Left cerebellar tumor
P11	Primary Large B-cell Lymphoma of the CNS	63	M	Left occipital
P12	Medulloblastoma, WHO grade 4	6 y	F	Left cerebellar tumor
P13	Anaplastic Meningioma, WHO grade 3	55	M	Left frontal
P14	Brain Tumor—Glioblastoma, WHO grade 4	51	F	Right frontal lobe
P15	Brain Tumor—Meningioma, WHO grade 1	55	F	Left temporal
P16	Brain Tumor—Glioblastoma, WHO grade 4	57	M	Right hemispheric lesion

**Table 5:** Cell culture media used for primary cells and their recipe.

Basic Growth Media	
Component	Volume for 500 mL
A DMEM	450 mL
FBS serum 10%	50 mL
Pen/Strep	5 mL
Glutamine	5 mL
Cell medium (1)	
DMEM/F-12 Complete	22.5 mL
DMEM ultamax Complete	22.5 mL
FBS serum 20%	5 mL
Gentamicin (50 µg/mL)	12.5 µL
ITS 0.1%	50 µL
EGF stock	50 µL
HEPES 1 M	550 µL
• EGF stock (1 µL in 100 µL H_2_O) 20 ng/mL	
Cell medium (2)	
DMEM/F-12 Complete	22.5 mL
DMEM gultamax Complete	22.5 mL
B-27	1 mL
Gentamicin (50 µg/mL)	12.5 µL
ITS 0.1%	50 µL
EGF stock	50 µL
HEPES 1 M	550 µL
Cell medium (3)	
NeuroCult™ Neural Cell Culture Media	Volume for 50 mL
BDNF (500 ng/mL)	25 µL
FBS serum 10%	5 mL
Pen/Strep	500 µL
Cell medium (4)	
Complete Advanced DMEM	Volume for 20 mL
Human Recombinant Brain-derived Neurotrophic Factor (BDNF) (500 ng/mL)	10 µL

## Data Availability

The original contributions presented in this study are included in the article/Supplementary Materials. Further inquiries can be directed to the corresponding author.

## Supplementary Materials

The following supporting information can be downloaded at https://www.mdpi.com/article/10.3390/ijms27167139/s1.
