# Supplementary material for: Saudi Patient-Derived Brain and Intracranial Explanted Tumor Cells: Isolation, Growth, and Anticancer Drug Screening
Source: Int J Mol Sci. 2026 Aug 9;27(16):7139. doi: 10.3390/ijms27167139 (PMC13513158; doi:10.3390/ijms27167139)
Supplement: Supplementary file 1 [file ijms-27-07139-s001.zip › ijms-4307323-supplementary.pdf]

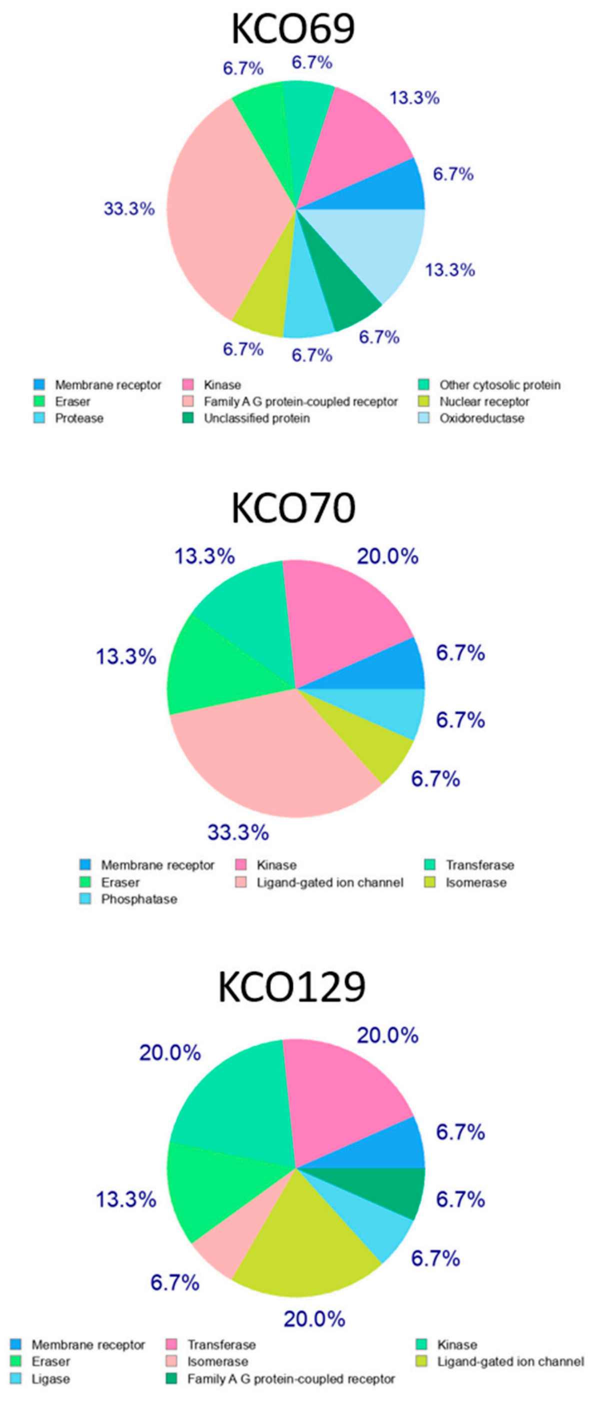

Supplementary Figure S1: A wheel graph of the target classes for the 3 compounds generated using Swiss Target Prediction
